# Tumor ADC value predicts outcome and yields refined prognostication in uterine cervical cancer

**DOI:** 10.1186/s40644-025-00828-6

**Published:** 2025-02-28

**Authors:** Njål Lura, Kari S. Wagner-Larsen, Stian Ryste, Kristine Fasmer, David Forsse, Jone Trovik, Mari K. Halle, Bjørn I. Bertelsen, Frank Riemer, Øyvind Salvesen, Kathrine Woie, Camilla Krakstad, Ingfrid S. Haldorsen

**Affiliations:** 1 Mohn Medical Imaging and Visualization Centre, Department of Radiology, Haukeland University Hospital, Jonas Lies Vei 65, 5021 Bergen, Norway; 2 Section for Radiology, Department of Clinical Medicine, University of Bergen, Bergen, Norway; 3 Department of Obstetrics and Gynecology, Haukeland University Hospital, Bergen, Norway; 4 Department of Clinical Science, Centre for Cancer Biomarkers, University of Bergen, Bergen, Norway; 5 Department of Pathology, Haukeland University Hospital, Bergen, Norway; 6 Neuro-SysMed, Department of Neurology, Haukeland University Hospital, Bergen, Norway; 7 Clinical Research Unit, Department of Clinical and Molecular Medicine, Norwegian University of Science and Technology, Trondheim, Norway

**Keywords:** DWI, Cervical cancer, ADC

## Abstract

Pelvic MRI is essential for evaluating local and regional tumor extent in uterine cervical cancer (CC). Tumor microstructure captured by diffusion-weighted imaging (DWI) and apparent diffusion coefficient (ADC) markers may be closely linked to prognosis in CC.

**Purpose** To explore whether primary tumor ADC markers predict survival in CC.

**Material and methods** CC patients (*n* = 179) diagnosed during 2009–2020 with MRI-assessed primary maximum tumor_size_ ≥ 2 cm were included in this retrospective single-center study. Two radiologists read all MRIs independently, measuring mean tumor ADC values in manually drawn regions of interest (ROIs) and mean tumor ADC (tumor_ADCmean_) from five measurements for the two readers was used. ADC from ROIs in the myometrium (myometrium_ADC_), cervical stroma (cervix_ADC_), and bladder (bladder_ADC_) were used to calculate ADC ratios. ADC markers were explored in relation to the International Federation of Gynecology and Obstetrics (FIGO) (2018) stage, disease-specific survival (DSS), and recurrence/progression-free survival (RPFS).

**Results** Inter-reader agreement for all ADC measurements was high (ICC:0.59–0.79). Low tumor_ADCmean_ predicted advanced FIGO stage (*P* = 0.04) and reduced DSS (hazard ratio (HR): 0.96, *P* < 0.001; AIC: 441). Myometrium_ADC_/tumor_ADCmean_ yielded the best Cox regression fit (AIC = 430) among all tumor ADC markers. Patients with high myometrium_ADC_/tumor_ADCmean_ had significantly reduced 5-year DSS for FIGO stage I, II, and III (*P* = 0.01, 0.004, and 0.02, respectively) and tended to the same for FIGO IV (*P* = 0.22).

**Conclusion** Low tumor_ADCmean_ predicted reduced DSS in CC. High myometrium_ADC_/tumor_ADCmean_ was the strongest ADC predictor of poor DSS and a marker of high-risk phenotype independent of FIGO stage.

**Supplementary Information:**

The online version contains supplementary material available at 10.1186/s40644-025-00828-6.

## Introduction

Uterine cervical cancer (CC) is the fourth most common cancer in women worldwide and one of the leading causes of cancer-related deaths [1]. CC patients are staged according to the International Federation of Gynecology and Obstetrics (FIGO) (2018) staging system [2]. Reported 5-year overall survival is high (~ 95%) in FIGO stage IA (micro-invasive disease), but drops to only 15% in FIGO stage IVB (distant spread) disease [3]. Pelvic magnetic resonance imaging (MRI) is widely used at primary diagnostic workup in CC, and MRI-derived information about tumor size, local tumor extent, and/or lymph node enlargement has since 2018 been incorporated in the FIGO stage assignment guiding therapeutic strategy.

Diffusion-weighted imaging (DWI) is also emerging as a valuable MRI technique, allowing the depiction of diffusion properties of the tissues, which can aid in distinguishing malignant from non-malignant tissue [4]. Malignant solid tumors are typically highly cellular, which impedes random Brownian movements of molecules and restricts water diffusion, whereas benign tissue typically has lower cell densities, allowing more free diffusion of water molecules. Tissue diffusion is quantified on the apparent diffusion coefficient (ADC) maps from DWI [5].

Low tumor ADC values have been reported to predict poor survival in gliomas and bladder cancer [6, 7]. Several smaller CC studies (cohorts of 42–85 patients) have also reported that low pre-treatment tumor ADC values predict shorter disease-free survival [8–11]. Furthermore, low tumor ADC has been found to predict pelvic lymph node metastasis in CC, including sub-centimeter lymph node metastases [12, 13]. Interestingly, while increasing tumor ADC values during radio-/chemotherapy has been linked to therapeutic response in CC [14, 15], a recent meta-analysis found that pre-treatment tumor ADC alone does not predict response to radio-/chemotherapy in CC patients [16]. However, ADC values are susceptible to significant variability depending on MRI protocol parameters, and relative ADC values have been suggested to be more robust than absolute ADC values for evaluating diffusion restriction [17]. Normalizing tumor ADC by ADC values in putative normal reference tissue (e.g. prostate, urine, or white matter of the brain) has been shown to improve prognostication by tumor ADC measurements in prostate [18], ovarian [19], brain [20] and uterine cervical [9] cancers, but is not performed in most studies on CC. Importantly, inter-reader variability for ADC measurements may impact the validity of tumor ADC as a potential imaging biomarker. Hence, the clinical utility of tumor ADC measurements to support pretherapeutic staging, prognostication, and response evaluation in CC treatment is not yet defined.

This study aims to assess inter-reader reproducibility for multiple ADC measurements, evaluate the impact of different MRI protocol parameters on measured tumor ADC values, and explore the value of different tumor ADC/normalized tumor ADC values for pre-treatment MRI staging and prognostication in CC.

## Methods

### Patients

**Fig. 1:**
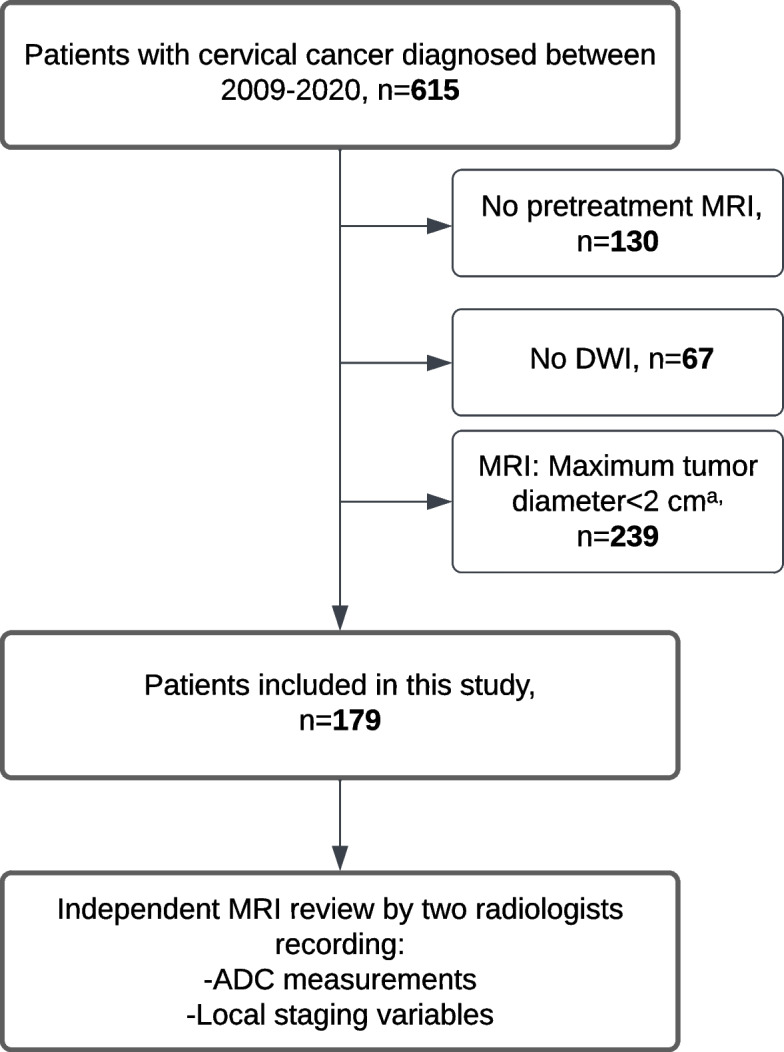
Flow chart illustrating patient inclusion and MRI review with ADC measurements and local tumor staging. primary radiotherapy with/without chemotherapy in 72% (130/179), and palliative chemotherapy/supportive. ADC, apparent diffusion coefficient; DWI, diffusion-weighted imaging; MRI, magnetic resonance imaging. ^a^ Only patients with MRI-assessed maximum tumor diameters ≥ 2 cm were included in order to allow reliable tumor-ADC measurements

This study was conducted with institutional review board (IRB) approval (2015/2333/REK Vest) and written informed consent from all patients. Pre-treatment MRI was available in 485 out of 615 (79%) histologically confirmed CC patients treated at the same university hospital (serving a population of ~ 1 million inhabitants) during 2009–2020. After excluding patients with MRI examinations without DWI (*n* = 67) and/or with MRI-assessed primary maximum tumor_size_ < 2 cm (*n* = 239), the final study cohort comprised 179 CC patients (Fig. 1). Patient data, including age, primary treatment, recurrence/progression-free survival (RPFS) and disease-specific survival (DSS), was collected from patient records (last accessed March 2023) and correspondence with the responsible gynecologist. The staging was conducted according to FIGO (2018), which allows the incorporation of imaging- and pathology findings in the stage assignment [2]. Histological type and grade were histopathologically assessed by an expert pathologist, as previously described [21]. The median (inter-quartile range [IQR]) follow-up time for all patients was 50 [24–80] months and 66 [37–87] months for survivors. In total, 26% (46/179) of the patients died from cervical cancer during follow-up; among these 91% (42/46) died within 5 years and 61% (28/46) within 18 months after primary diagnosis. Among patients with FIGO stage ≤ IVA (*n* = 160), 25% (40/160) experienced disease recurrence or progression (RPFS) at a median [IQR] of 12 [7–23] months from primary diagnosis. Their sites of recurrence were local pelvic (*n* = 16), locoregional with lymph node involvement (*n* = 3), and distant sites (*n* = 21). Subsequently, 68% (27/40) of these patients succumbed to cervical cancer within a median (IQR) of 5 (3-11) months after recurrence. Primary treatment (before recurrence) in these patients included surgery alone (*n* = 2), surgery with adjuvant therapy (*n* = 6), and primary radiotherapy with or without chemotherapy (*n* = 32). At recurrence, treatments provided were radiotherapy (*n* = 17), chemotherapy (*n* = 15), combined therapy (*n* = 3), and palliative care (*n* = 5).

### MRI Protocol

The MRI examinations were performed with scanners from Siemens Healthineers (Erlangen, Germany)/GE Healthcare (Waukesha, WI)/Philips Healthcare (Eindhoven, Netherlands) in 118/49/12 patients and on 1.5 T and 3.0 T systems in 56% (100/179) and 44% (79/179), respectively. The imaging protocols included pelvic sagittal and axial oblique (perpendicular to the long axis of the uterine cervix) T2-weighted images in all and axial non-contrast [contrast-enhanced] T1-weighted gradient-echo images without fat suppression in 99% (178/179) [14% (25/179)] of the examinations. All MRI examinations included a diffusion-weighted sequence in axial oblique/axial (*n* = 179) and/or sagittal (*n* = 49) planes with the highest b-values of 800 (*n* = 57) and 1000 (*n* = 122) and lowest b-values of 0 (*n* = 104) and 50 (*n* = 75). The median (IQR) echo time (TE) was 71 (65–82) milliseconds, and the median (IQR) repetition time (TR) was 3600 (3100–5640) milliseconds (Suppl Table 1).

### ADC measurements

**Fig. 2:**
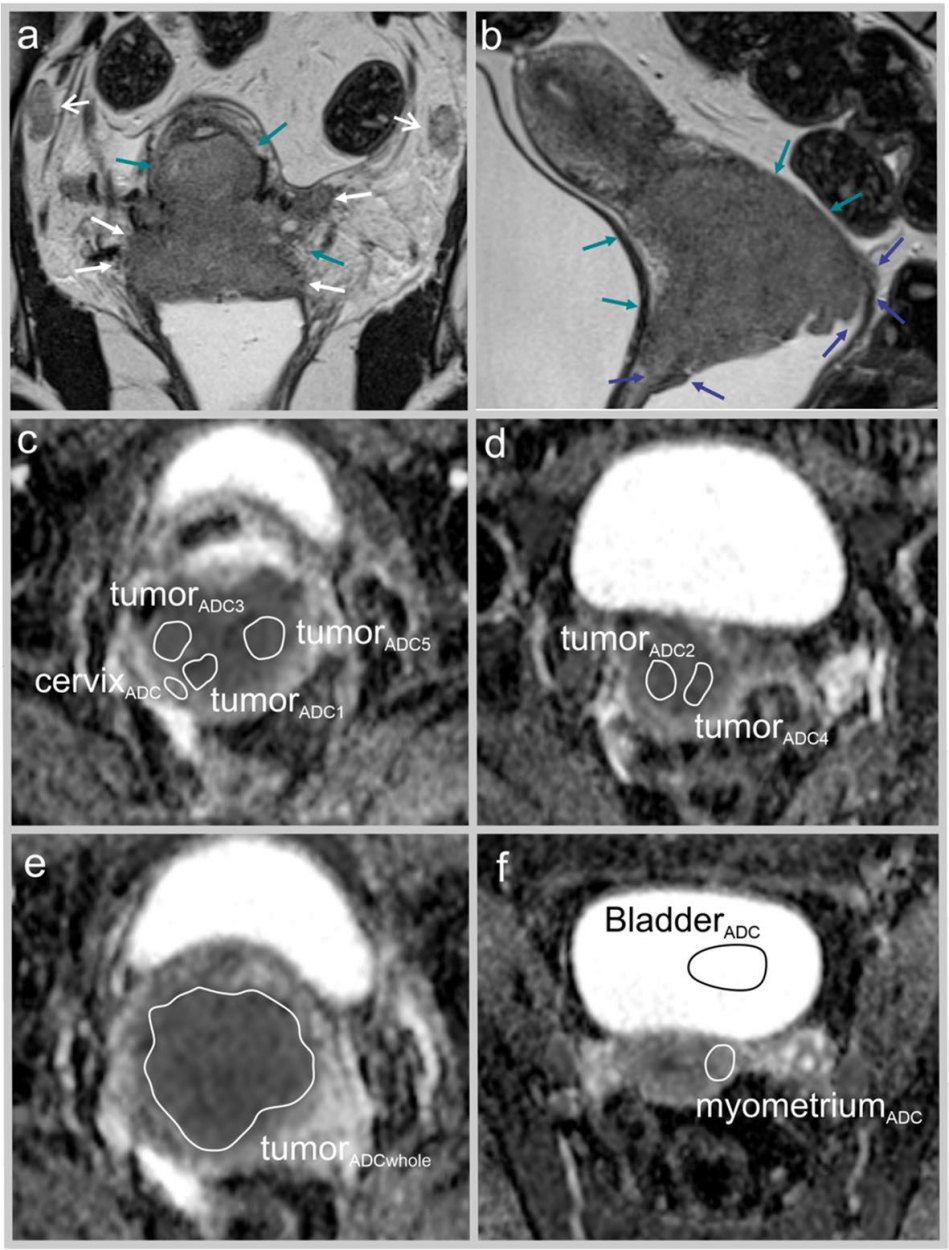
MRI in a 30-year-old woman presenting with a large cervical tumor (squamous cell carcinoma, FIGO (2018) stage IIIC1) prior to treatment with concomitant radio- and chemotherapy. This patient had low myometrium_ADC_/tumor_ADCmean_-ratio at primary diagnosis and she had no signs of recurrence after 8.5 years. Coronal (**a**) and sagittal (**b**) T2-weighted MRI depicts a large cervical tumor (green arrows; with maximum diameter 8.5 cm) and disrupted stromal ring (white arrows) (**a**,**b**), tumor growth into the upper 2/3 of the vagina (blue arrows) (**b**) and enlarged (short axis diameter > 1 cm) iliac lymph nodes (open white arrows) (**a**). Axial oblique (relative to the long axis of the cervix) ADC-maps (**c**–**f**) depict restricted diffusion in the primary tumor. The following regions of interest were drawn on the ADC maps: tumorADC_1–5_, tumor_ADCwhole_ bladder_ADC_, cervix_ADC_ and myometrium_ADC._ Tumor_ADCmean_ was calculated as the mean of tumor_ADC1–5_, derived by two independent readers

**Fig. 3:**
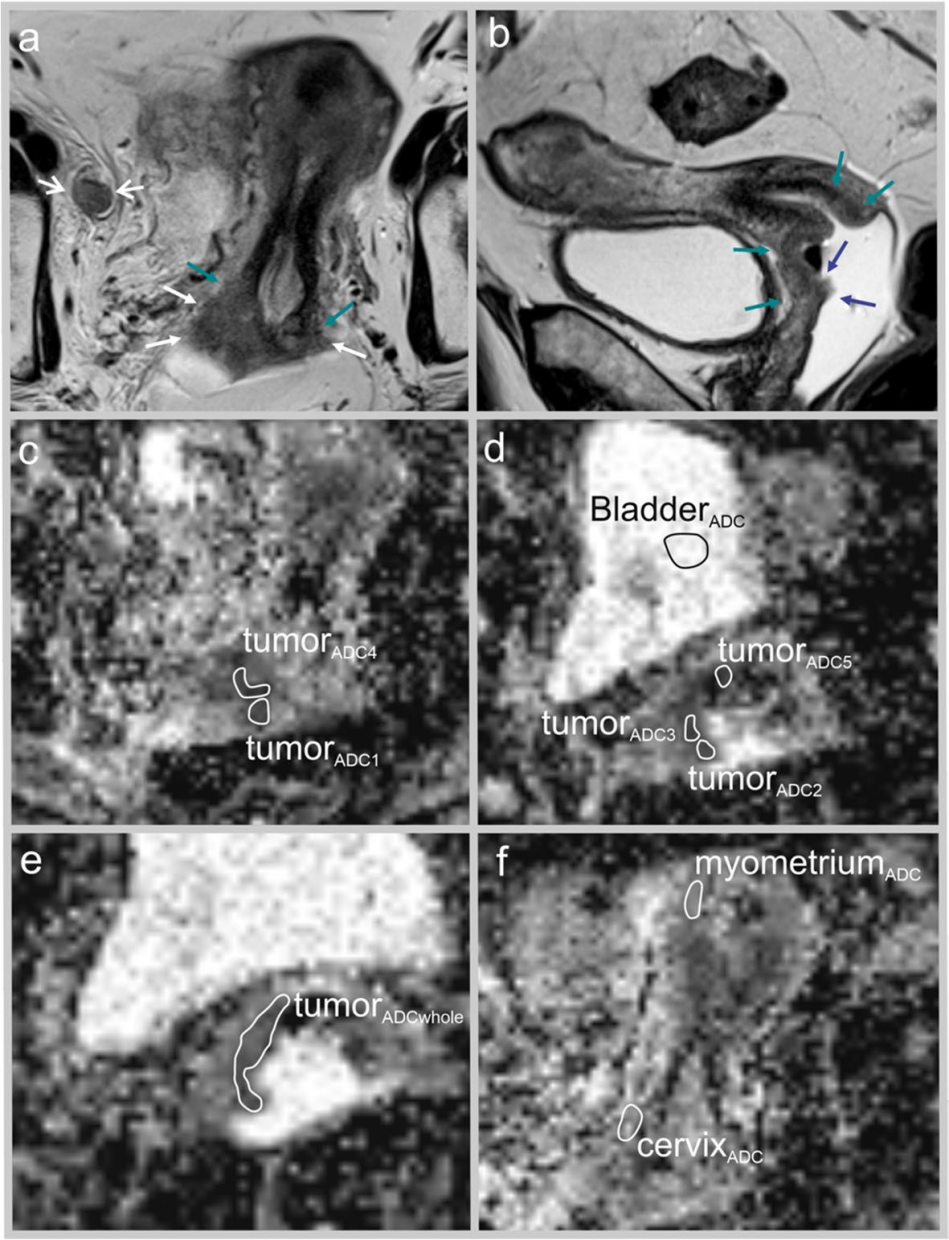
MRI in a 66-year-old woman presenting with a moderately large cervical tumor (squamous cell carcinoma, FIGO (2018) stage IIIC1) prior to treatment with concomitant radio- and chemotherapy. This patient had high myometrium_ADC_/tumor_ADCmean_-ratio and eventually died from cervical cancer 4.5 years after primary diagnosis. Axial oblique (relative to the long axis of the cervix) (**a**) and sagittal (**b**) T2-weighted MRI depicts an infiltrative moderately large (maximum tumor diameter of 3.9 cm) tumor (green arrows; a,b)in the uterine cervix with disrupted stromal ring to the right indicating parametrial invasion (white arrows; a), tumor growth into the upper 2/3 of the vagina (blue arrows; b) and enlarged right-sided iliac lymph node (white open arrows; a). Para-axial ADC-maps (**c**-**f**) depict restricted diffusion in the primary tumor. Regions of interest were drawn on the the ADC maps: tumor_ADC1–5_, tumor_ADCwhole_ bladder_ADC_, cervix_ADC_ and myometrium_ADC_ and tumor_ADCmean_ was calculated as the mean of tumor_ADC1–5_, derived by two independent readers

All images were de-identified and read independently by readers who were blinded for clinical data, histological diagnosis, and patient outcome. Two readers (with 7 years [reader 1, NL] and 3 years [reader 2, SR] of experience) drew regions of interest (ROIs) on the axial oblique/axial (ADC maps by using the polygon drawing function in the PACS software (Sectra, Lindköping, Sweden) yielding mean ROI ADC values and ROI areas for all patients (*n* = 179). One ROI (tumor_ADCwhole_) comprising the entire primary tumor on the axial slice depicting the largest tumor area was drawn. Furthermore, five tumor ADC ROIs (tumor_ADC1–5_) were drawn, intentionally selecting the tumor areas with the visually evaluated lowest ADC values on the ADC maps. Mean tumor ADC (tumor_ADCmean_) was calculated as the mean of tumor_ADC1–5_. All tumor ROIs were carefully drawn, aiming to avoid areas that appeared fluid-rich or necrotic (Figs. 2 and 3). A variable simulating the ADC value if only using a single ROI (tumor_ADCrandom_) was computed by random sampling from the five ADC measurements (tumor_ADC1–5_) by the two readers. The mean of the ADC variables from readers 1 and 2 was calculated and used in further analysis.

In addition, in order to reduce ADC variability caused by variations in MRI protocols, ROIs were drawn in putative normal reference tissue, i.e., urine in the urinary bladder (bladder_ADC_), using a similar approach as Gladwish et al. [9], normal outer cervical stroma (cervix_ADC_), and normal myometrium (myometrium_ADC_) in order to compute normalized tumor ADC values representing ratios of tumor_ADCmean_ and myometrium_ADC_/cervix_ADC_/bladder_ADC_ (Figs. 2 and 3). Ratios with tumor_ADCmean_ in the numerator and in the denominator, and log-transformed ratios were evaluated. All of the derived ADC ratios were compared in further statistical analyses.

### MRI-derived local staging variables

MRI-based local staging parameters (maximum tumor diameter, tumor invasion into the vagina/parametrium/urinary bladder/ rectum, and enlarged [> 1.0 cm short-axis diameter] pelvic lymph nodes) were derived from three independent readings by four radiologists having 3–20 years of experience with pelvic MRI (Two radiologists reading all imaging examinations (*n* = 179) and two different radiologists reading 79 and 120 examinations, respectively). Consensus variables for MRI staging parameters were based on majority vote (for categorical variables) and median values (for tumor size).

### Statistical methods

Tumor ADC variables (including ratios) were analyzed in relation to clinicopathologic patient characteristics, MRI scanning protocol (e.g., field strength, acquisition parameters), and MRI staging findings using Mann–Whitney U test, Kruskal–Wallis H test, or Jonckheere–Terpstra trend test and linear regression: Non-parametric tests were used since not all variables were normally distributed. However, the residuals from all linear regression models satisfied assumptions of normal distribution (Shapiro Wilks test, all *P* > 0.05). The correlation between tumor ADC measurements was assessed using Spearman’s rank correlation test.

Inter-reader agreement for ADC measurements was assessed by intraclass correlation coefficient ICC analysis and classified as poor (ICC = 0–0.39), fair (0.40–0.59), good (0.60–0.74), or excellent (0.75–1.00) [22].

Cox regression and Kaplan–Meier curves were used for survival analysis. All variables in the Cox regression analyses satisfied the assumption of proportional hazard (the Schoenfeld test of residuals, *P* ≥ 0.09), with the exception of FIGO stage (I-IV), which was included as a stratifying variable. Cox regression fit for predicting DSS was evaluated using the Akaike information criterion (AIC) method; a difference of > 2 in AIC between two models indicates a preference for the model yielding the lowest AIC [23]. The “fastbw” model selection algorithm from the “rms” R-package [24] was used for multivariable Cox model selection. Multiple imputations were performed using the “mice”-algorithm [25] for missing values in Cox regression analysis, as this is preferred over complete case analysis (only including cases with no missing values) [26].

Time-dependent receiver operating characteristics curve (tdROC) analyses, assessing how well a diagnostic model predicts time-dependent outcomes [27], were used to assess and compare the performance of the ADC measurements, and Cox models were used for predicting DSS. The area under the curve integrated over 5 years after diagnosis (iAUC) was estimated using the “RisksetROC” R-package [28], and the time-dependent area under the curve at 3 years after diagnosis was estimated using the “timeROC” R-package [29]. Comparisons of tdROCs and evaluation of optimism bias [30] were performed using bootstrapping with resampling 10,000 times, and no significant optimism bias was found in any of the ROC analyses (P ≥ 0.26). The tdROC curve determined optimal cut-offs for ADC variables for predicting DSS within FIGO subgroups 5 years after diagnosis, selecting the highest Youden index [31]. To estimate the hazard ratio, P-value, and AIC for tumor_ADCrandom_, 10,000 iterations of random sampling were used, and mean values with 95% confidence intervals were calculated. The data were analyzed using R software (version 4.0.3, R Foundation for Statistical Computing, Vienna, Austria) [32]. All reported P-values were two-sided and considered significant when below 0.05.

## Results

### Patients and treatment

The median (IQR) patient age at primary diagnosis was 49 (39–62) years in the final patient cohort (*n* = 179). In total, 28% (50/179) were diagnosed with FIGO stage I, 22% (39/179) with stage II, 37% (66/179) with stage III, and 13% (24/179) with stage IV (Table 1). Primary treatment consisted of surgery only in 15% (27/179), surgery with adjuvant therapy in 10% (17/179), primary radiotherapy with or without chemotherapy (RCT) in 72% (129/179), and palliative chemotherapy/supportive care in 3% (6/179).

**Table 1:** Tumor_ADCmean_ values in relation to FIGO (2018) stage, MRI-assessed staging parameters and histologic subtype and grade

Variable	N: 179 (100%)	Tumor_ADCmean_ (10^–6^ mm^2^/sec) [IQR]	*P*-value
**FIGO (2018) stage**			0.04
I	50 (28%)	767 [661–856]	
II	39 (22%)	745 [648–836]	
III	66 (37%)	735 [651–814]	
IV	24 (13%)	709 [622–805]	
**Vaginal invasion**			0.04
No	49 (28%)	776 [679–857]	
Upper two thirds	119 (67%)	731 [641–822]	
Lower third	11 (6%)	719 [627–776]	
**Parametrial invasion**			0.03
No	43 (24%)	776 [673–897]	
Yes	136 (76%)	726 [635–819]	
**Enlarged (> 1 cm) lymph nodes**			0.20
No	137 (77%)	741 [647–840]	
Yes	42 (23%)	728 [630–796]	
**Invasion into rectum/urinary bladder**			0.91
No	134 (75%)	744 [643–841]	
Yes	45 (25%)	733 [664–813]	
**Histologic subtype**			0.16
Adenocarcinoma	33 (18%)	760 [646–865]	
Squamous cell carcinoma	139 (78%)	733 [643–829]	
Other^a^	7 (4%)	619 [515–735]	
**Histologic grade**			< 0.001
Low/moderate grade	129 (72%)	752 [653–834]	
High grade	31 (17%)	679 [547–735]	
Missing	19 (11%)	798 [737–892]	
**Tumor**_**ADCmean**_** as dependent variable**^b^			
**Variable**	**R**^**2**^	**β**	**P**
**Age (pr. decade) (*n* = 179), median (IQR): 49 (39–62) yrs**	0.00	-0.52	0.94
**MRI: Maximum tumor diameter (*n* = 179), median (IQR): 4.8 (3.5–6.2) cm**	0.02	-6.93	0.08

*P*-values represent the difference in tumor_ADCmean_ across groups, estimated by Mann Whitney U test and by Kruskal Wallis test, Joncheere Terpsta trend test for multiple categories, or a significant association between tumor_ADCmean_ and an independent variable in linear regression

*ADC* apparent diffusion coefficient, *IQR* inter-quartile range, *FIGO* International Federation of Gynecology and Obstetrics

^a^ Neuroendocrine (*n* = 4), undifferentiated (*n* = 2) and adenosquamous (*n* = 1) tumors

^b^ Linear regression analysis for continuous variables with tumor_ADCmean_ as the dependent variable

### Inter-reader agreement for ADC measurements

The inter-reader agreement for measuring tumor_ADC1-5_, tumor_ADCmean_, tumor_ADCwhole_ was good to excellent (ICC: 0.67–0.78) (Suppl. table 2). Inter-reader agreement for measuring bladder_ADC_, cervix_ADC_, and myometrium_ADC_ was excellent (ICC: 0.79), fair (ICC: 0.59), and good (ICC: 0.64), respectively. The ratios bladder_ADC_**/**tumor_ADCmean_, cervix_ADC_**/**tumor_ADCmean_, and myometrium_ADC_/tumor_ADCmean_ all had a good inter-reader agreement (ICC: 0.67–0.74; Suppl. table 2). The ADC variables generated from readers 1 and 2 yielded comparable prognostic power in subsequent survival analyses, suggesting good inter-reader agreement for extracting prognostic ADC markers (Suppl. table 3).

### Tumor ADC and clinicopathologic patient features

The median [IQR] ADC values for tumor_ADC1–5_, tumor_ADCmean_ and tumor_ADCwhole_ ranged from 682 [572–777]—866 [766–952] 10^–6^ mm^2^/sec, being significantly lower than the ADC values for bladder_ADC_ (2930 [2603–3169] 10^–6^ mm^2^/sec), cervix_ADC_ (1631 [1411–1760] 10^–6^ mm^2^/sec) and myometrium_ADC_ (1412 [1258–1564] 10^–6^ mm^2^/sec) (*P* < 0.001) (Table 2). All tumor ADC measurements were strongly positively correlated (Spearman’s rho: 0.83–0.99, *P* < 0.001). Furthermore, significant but less pronounced positive correlations were observed between tumor ADC and ADC in the reference tissue (bladder_ADC_, cervix_ADC,_ and myometrium_ADC_; Spearman’s rho ≤ 0.36, *P* < 0.001; Table 2).

**Table 2:** Median (IQR) apparent diffusion coefficient (ADC) values in primary tumor and normal pelvic reference tissue and their correlations in 179 patients with cervical cancer

	Tumor_ADC1_^a^	Tumor_ADC2_^a^	Tumor_ADC3_^a^	Tumor_ADC4_^a^	Tumor_ADC5_^a^	Tumor_ADCmean_^a^	Tumor_ADCwhole_^a^	Bladder_ADC_^a^	Cervix_ADC_^a^	Myometrium_ADC_^a^
Median ADC (IQR) ${(10}^{-6} {\text{mm}}^{2}/\text{s})$	682 (572–777)	715 (616–813)	740 (650–835)	767 (673–867)	794 (709–892)	736 (644–831)	866 (766–952)	2930^b^ (2603–3169)	1631^b^ (1411–1760)	1412^b^ (1258–1564)
	r	r	r	r	r	r	r	r	r	r
Tumor_ADC1_	1	0.98*	0.96*	0.95*	0.93*	0.98*	0.83*	0.36*	0.30*	0.30*
Tumor_ADC2_		1	0.98*	0.97*	0.95*	0.99*	0.84*	0.35*	0.31*	0.30*
Tumor_ADC3_			1	0.98*	0.97*	0.99*	0.86*	0.35*	0.30*	0.31*
Tumor_ADC4_				1	0.98	0.99*	0.85*	0.35*	0.30*	0.32*
Tumor_ADC5_					1	0.98*	0.85*	0.32*	0.25*	0.28*
Tumor_ADCmean_						1	0.86*	0.35*	0.29*	0.31*
Tumor_ADCwhole_							1	0.43*	0.32*	0.35*
Bladder_ADC_								1	0.55*	0.75*
Cervix_ADC_									1	0.49*
Myometrium_ADC_										1

*ADC* apparent diffusion coefficient, *Tumor*_*ADC1–5*_ tumor-ADC values measured in five regions of interest (ROIs) in primary tumor (presented in ascending order: tumor_ADC1_ < tumor _ADC2_ etc.), selecting the areas depicting most restricted diffusion; tumor_ADCmean_, mean value of tumor_ADC1_–tumor_ADC5_; tumor_ADCwhole_, ADC value from a ROI comprising the entire primary tumor in the slice depicting largest tumor area; Bladder_ADC_, ADC values in ROI comprising urine in the urinary bladder; Cervix_ADC_**,** ADC values measured in the normal outer cervical stroma; myometrium_ADC_, ADC values measured in the normal myometrium; *CI* confidence interval, *ICC* intra-class correlation, *IQR* inter-quartile range

*r* = Spearman’s rank correlation coefficient (rho)

^*^Correlation is significant, *P* < 0.001 (2-tailed)

^a^ ADC variables are consensus variables calculated as the mean value from two readers

^b^ The ADC value is significantly higher than all the tumor ADC values (*P* < 0.001, Mann Whitney U test)

Low tumor_ADCmean_ and high myometrium_ADC_/tumor_ADCmean_ were significantly more common in patients with advanced FIGO stage, high-grade histology, and vaginal- or parametrial tumor invasion (*P* ≤ 0.04; Table 1 and Suppl. table 4). Low tumor_ADCmean_ was not associated with patient age, MRI-assessed primary maximum tumor_size_, rectal-/bladder invasion, enlarged (> 1.0 cm) pelvic lymph nodes, or histological subtype (*P* ≥ 0.08 for all; Table 1). However, high myometrium_ADC_/tumor_ADCmean_ was significantly associated with large primary maximum tumor_size_ (*P* < 0.001) and enlarged pelvic lymph nodes (*P* = 0.02; Suppl. table 4).

### Impact of DWI-MRI protocol parameters on tumor ADC values

Patients scanned on 3.0 T MRI had higher tumor_ADCmean_ (median [IQR]:774 [712–864] 10^–6^ mm^2^/sec) than patients scanned on 1.5 T (median [IQR]: 702 [629–810]) 10^–6^ mm^2^/sec (*P* = 0.002, R^2^ = 0.05; Suppl. Table 5). Low lowest b-value (0 vs. 50) were associated with higher tumor_ADCmean_ (b-value of 0: median [IQR]: 749 [666–852] 10^–6^ mm^2^/sec vs. b-value of 50: 730 [632–819] 10^–6^ mm^2^/sec; R^2^ = 0.04, *P* = 0.009). Also, high repetition time was associated with higher tumor_ADCmean_ (R^2^ = 0.03, *P* = 0.02; Suppl. Table 5). MRI echo time, FOV, matrix dimension, slice thickness, inter-slice gap, vendor (Siemens Healthineers/Philips Healthcare /GE Healthcare), number of b-values (2, 3 or 4) or highest b-value (1000 vs. 800 s/mm^2^) were not associated with tumor_ADCmean_ (*P* ≥ 0.07). Furthermore, higher values for tumor_ADCmean_ were associated with higher bladder_ADC_, cervix_ADC_, and myometrium_ADC_ (R^2^ of 0.10, 0.10, and 0.14, respectively; *P* < 0.001). In the multiple linear regression analysis (including flip angle, field strength, repetition time, lowest b-value, bladder_ADC_, cervix_ADC,_ and myometrium_ADC_), only myometrium_ADC_ was independently associated with tumor_ADCmean_ (*P* = 0.01; Suppl. table 5). No MRI protocol parameters significantly correlated to myometrium_ADC_/tumor_ADCmean_ ( *P* ≥ 0.05 for all).

### Low tumor ADC predicts poor survival

Low tumor ADC for all the tumor ADC variables predict poor disease-specific survival (DSS) with hazard ratios of 0.96–0.98 per 10 unit increase in ADC value (10^–6^ mm^2^/sec; *P* ≤ 0.02) and all tumor ADC variables yield similar Cox regression fit (AIC: 441–443), except for tumorADC_whole_ (AIC: 449) and tumor_ADCrandom_ (AIC: 444) yielding slightly inferior fit compared to tumorADC_mean_ (AIC: 441; Table 3). Normalizing tumor_ADCmean_ to bladder_ADC_, cervix_ADC_, and myometrium_ADC_ by computing ADC ratios yielded better Cox regression fit when using tumor_ADCmean_ as denominator rather than numerator (Table 3). Among the ratios, myometrium_ADC_/tumor_ADCmean_ yielded the best Cox regression fit (AIC: 430) and was hence selected for further multivariable analyses (Table 4). Univariable analyses also showed that large MRI-derived maximum tumor_size_ (cm), MRI-assessed tumor invasion into the vagina/bladder/rectum, enlarged (> 1.0 cm) lymph nodes, high age (decade) and high-grade tumor predicted reduced DSS (hazard ratios: 1.28–5.26, *P* ≤ 0.009), whereas parametrial infiltration did not (hazard ratio = 1.89, *P* = 0.12; Table 3).

**Table 3:** Primary tumor-ADC measurements, tumor-ADC normalized by reference tissue and clinicopatologic/MRI markers for predicting disease-specific survival (DSS) in cervical cancer (*n* = 179; 46 died from disease)

	**HR**	**95%CI**	**P**^a^	**AIC**
**Primary tumor-ADC variables**				
Tumor_ADCwhole_	0.98	0.96–1.00	0.02	449
Tumor_ADCrandom_^b^	0.97	0.96–0.98	0.003	444
Tumor_ADC1_	0.97	0.95–0.98	0.001	443
Tumor_ADC2_	0.96	0.94–0.98	< 0.001	441
Tumor_ADC3_	0.96	0.94–0.98	< 0.001	442
Tumor_ADC4_	0.96	0.94–0.98	< 0.001	441
Tumor_ADC5_	0.96	0.94–0.99	< 0.001	441
Tumor_ADCmean_^c^	0.96	0.94–0.98	< 0.001	441
**Normalized tumor-ADC variables**				
Tumor_ADCmean_/bladder_ADC_	0.30	0.17–0.56	< 0.001	438
Tumor_ADCmean_/cervix_ADC_	0.60	0.43–0.84	0.003	444
Tumor_ADCmean_/myometrium_ADC_	0.51	0.38–0.70	< 0.001	434
Log (tumor_ADCmean_/bladder_ADC_)^d^	0.06	0.02–0.22	< 0.001	437
Log (tumor_ADCmean_/cervix_ADC_)^d^	0.07	0.02–0.27	< 0.001	440
Log (tumor_ADCmean_/myometrium_ADC_)^d^	0.03	0.01–0.13	< 0.001	432
Bladder_ADC_/tumor_ADCmean_	1.82	1.42–2.34	< 0.001	437
Cervix_ADC_/tumor_ADCmean_	2.91	1.87–4.54	< 0.001	437
Myometrium_ADC_/tumor_ADCmean_^e^	4.64	2.68–8.04	< 0.001	430
**Clinical/histological/MRI variables**				
MRI:maximum tumor_size_ (cm)	1.34	1.23–1.47	< 0.001	423
MRI:invasion vagina^f^	5.26	2.70–10.0	< 0.001	428
MRI:parametrial infiltration (yes/no)	1.89	0.85–4.24	0.12	451
MRI:enlarged (> 1 cm) lymph nodes (yes/no)	2.45	1.35–4.42	0.003	446
MRI:invasion bladder/rectum (yes/no)	3.15	1.71–5.77	0.001	442
Histologic grade (low/moderate vs.high)^g^	2.90	1.58–5.32	< 0.001	444
Age (decade)	1.28	1.06–1.53	0.009	448

*ADC* apparent diffusion coefficient (10^−6^mm^2^/sec**)**, *AIC* Akaike information criterion, *CI* confidence interval, *HR* hazard ratio

^a^ *P*-value refers to Cox regression analysis of the variables’ relation to disease-specific survival

^b^ Tumor_ADCrandom_ was calculated by randomly sampling among Tumor_ADC1_–Tumor_ADC5_. To estimate HR, P-value and AIC, we performed 10000 iterations of the random sampling

^c^ Tumor_ADCmean_ was among the tumor-ADC variables with lowest AIC and was selected for calculating normalized tumor-ADC variables

^d^ We only present results for Log (tumor_ADCmean_/”normal tissue”) and not for Log (”normal tissue”/ tumor_ADCmean_) since these yield the same regression fit

^e^ We used myometrium_ADC_/tumor_ADCmean_ as the normalized variable in the multivariable analysis, as it yielded lowest AIC value among the tumor-ADC variables

^f^ Ordinal variable: “no invasion”, "invasion down to upper 2/3 of the vagina" and "invasion down to lower 1/3 of the vagina"

^g^ Multiple imputation were performed for missing values in Cox regression analysis; histologic grade information was missing in 19/179 patients

**Table 4:** Multivariable Cox regression analysis including clinicopathologic- and MRI variables for predicting disease-specific survival in 179 patients with uterine cervical cancer (46 died from disease)

	**HR**	**95%CI**	**P**^**b**^
**Multivariable model**^**a**^			
Myometrium_ADC_/tumor_ADCmean_	4.64	2.68–8.04	0.001
MRI: maximum tumor_size_ (cm)	1.34	1.23–1.47	< 0.001
MRI: invasion vagina^c^	5.26	1.58–5.32	0.004
Histologic grade (low/moderate vs.high)^d^	2.90	2.70–10.0	0.01
**Multivariable model**^**a**^** stratified by FIGO (2018) stages I-IV**			
Myometrium_ADC_/tumor_ADCmean_	2.43	1.45–4.88	0.006
MRI: maximum tumor_size_ (cm)	1.12	1.06–1.29	0.04
MRI: invasion vagina^c^	1.31	0.91–4.46	0.41
Histologic grade (low/moderate vs.high)^d^	2.79	1.19–4.99	0.002
**Myometrium**_**ADC**_**/tumor**_**ADCmean**_** in FIGO (2018) stages I-IV**			
FIGO stage I (*n* = 50, events = 3)^e^	26.8	2.03–354	0.01^f^
FIGO stage II (*n* = 39, events = 4)	21.5	2.62–176	0.004^f^
FIGO stage III (*n* = 66, events = 20)	4.01	1.15–14.3	0.02 ^f^
FIGO stage IV (*n* = 24, events = 19)	1.68	0.73–3.85	0.22
**Myometrium**_**ADC**_**/tumor**_**ADCmean**_** in treatment groups**			
Surgery only^g^ (*n* = 27, events = 0)	-	-	-
Surgery & adjuvant therapy (*n* = 17 events = 6)	3.66	1.13–11.85	0.03
RCT (*n* = 129, events = 32)	3.96	1.76–8.92	< 0.001
palliative care^g^ (*n* = 6, events = 6)	-	-	-

*ADC* apparent diffusion coefficient (10^−6^mm^2^/sec**)**, *AIC* Akaike information criterion, *CI* confidence interval, *FIGO* International Federation of Gynecology and Obstetrics, *HR* hazard ratio, *RCT* Primary radiotherapy with or without chemotherapy

^a^Variables in the model were selected by using the "fastbw"-algorithm in the "rms" r-package including the variables: Myometrium_ADC_/tumor_ADCmean_, MRI: maximum tumor_size_, MRI: parametrial infiltration (yes/no), MRI: enlarged (> 1 cm) lymph nodes (yes/no), MRI: invasion vagina, MRI: invasion bladder/rectum (yes/no), histologic grade, and age in the selection procedure

^b^ Cox regression analysis

^c^ Ordinal variable consist of "no invasion", "invading upper 2/3 of the vagina" and "invading lower 1/3 of the vagina"

^d^ Missing data were handled by multiple imputation in order to perform multivariable analysis on all patients in the sample. Data on histologic grade was missing in 11% (19/179) of the patients

^e^ The study cohort included only patients with MRI derived maximum tumor size ≥ 2 cm, consequently there were only FIGO stage ≥ 1B2 in the analysis in FIGO stage I

^f^ Myometrium_ADC_/tumor_ADCmean_ remained significant also after adjusting for MRI: maximum tumor_size_ in FIGO (2018) I, II and III (*P* = 0.02, *P* = 0.01 and *P* = 0.04, respectively)

^g^ Hazard ratios could not be estimated due to lack of events or survivors in this group

### Myometrium_ADC_/tumor_ADCmean_ is an independent predictor of disease-specific and recurrent/progression-free survival

In multivariable Cox analyses, myometrium_ADC_/tumor_ADCmean_, MRI-derived maximum tumor_size_, MRI-assessed vaginal tumor invasion, and histological grade were all identified as independent predictors of DSS (hazard ratios: 1.34–4.64, *P* ≤ 0.01; Table 4). After stratifying for FIGO stage (I–IV), myometrium_ADC_/tumor_ADCmean_, maximum tumor_size_ (cm), and histological tumor grade remained significant predictors of DSS (hazard ratios: 1.12–2.79, *P* ≤ 0.04), whereas vaginal tumor invasion did not (hazard ratio = 1.31, *P* = 0.41; Table 4).

In a subgroup analysis for FIGO stages I, II-, III- and IV patients, myometrium_ADC_/tumor_ADCmean_ significantly predicted DSS for FIGO I (hazard ratio = 26.8, *P* = 0.01), FIGO II (hazard ratio = 21.5, *P* = 0.004) and FIGO III (hazard ratio = 4.01, *P* = 0.02), and tended to the same for FIGO IV (hazard ratio = 1.68, *P* = 0.22; Table 4). Also, after adjusting for MRI-derived maximum tumor_size_, myometrium_ADC_/tumor_ADCmean_ remained a significant predictor of DSS FIGO I, FIGO II, and FIGO III (*P* = 0.02, *P* = 0.01, and *P* = 0.04, respectively). Moreover, high myometrium_ADC_/tumor_ADCmean_ also predicted reduced DSS in patients receiving surgery with adjuvant therapy (*n* = 17, HR = 3.76, *P* = 0.03) and in patients receiving RCT (*n* = 129, HR = 4.10, *P* < 0.001) (Table 4). For patients with FIGO stages ≤ IVA (*n* = 160), high myometrium_ADC_/tumor_ADCmean_ independently predicted reduced recurrence/progression-free survival (RPFS) after adjusting for FIGO stage (I-IV)(Suppl. table 6).

### Myometrium_ADC_/tumor_ADCmean_ combined with FIGO stage yield better prediction of survival

**Fig. 4:**
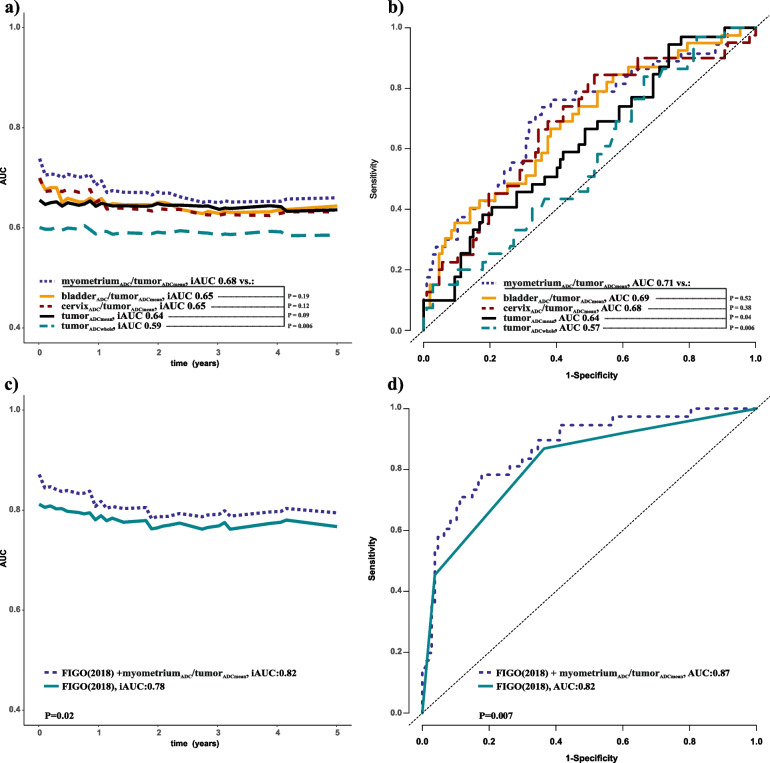
Time-dependent receiver operating characteristic (tdROC) analysis with iAUC at 5 years (**a**,**c**) and AUC at 3 years (**b**,**d**) after diagnosis for predicting disease-specific survival (DSS) in uterine cervical cancer (CC). Normalizing tumor_ADCmean_ to myometrium_ADC_ by calculating a myometrium_ADC_/tumor_ADCmean_ ratio yielded higher iAUC than for tumor_ADCwhole_ (iAUC: 0.68 vs.0.59, *P* = 0.006), and tended to yield higher iAUC than for tumor_ADCmean_ (iAUC: 0.68 vs.0.64, *P* = 0.09) (**a**). The tdROC-AUC at 3 years was higher for myometrium_ADC_/tumor_ADCmean_ than for tumor_ADCwhole_ and tumor_ADCmean_ (AUC at 3 years: 0.71 vs. 0.57 and 0.64, respectively)(**b**). Myometrium_ADC_/tumor_ADCmean_, bladder_ADC_**/**tumor_ADCmean_ and cervix_ADC_**/**tumor_ADCmean_ yielded similar iAUCs and AUCs (*P* ≥ 0.12). The myometrium_ADC_**/**tumor_ADCmean_ combined with FIGO (2018) yielded higher discriminatory performance for predicting DSS than FIGO (2018) alone (iAUC: 0.82 vs. 0.78, *P* = 0.02; AUC: 0.87 vs.0.82, *P* = 0.007) (**c**, **d**). ADC, apparent diffusion coefficient (10^−6^mm^2^/sec**)**; FIGO, International Federation of Gynecology and Obstetrics; iAUC, the integrated area under the curve for the specified time interval; tumor-ADC, ADC measurements from the primary tumor

Myometrium_ADC_/tumor_ADCmean_ yielded higher iAUC than tumor_ADCwhole_ for predicting 5-year DSS (iAUC: 0.68 vs. 0.59, *P* = 0.006), but similar iAUC to that of bladder_ADC_/tumor_ADCmean_, cervix_ADC_/tumor_ADCmean_ and tumor_ADCmean_ (iAUC: 0.68 vs. 0.65, 0.65 and 0.64, respectively; *P* ≥ 0.09) (Fig. 4a). Also, myometrium_ADC_/tumor_ADCmean_ yielded higher AUC than tumor_ADCwhole_ and tumor_ADCmean_ for predicting 3-year DSS (AUC: 0.71 vs. 0.57 and 0.64, respectively, *P* ≤ 0.04), but similar AUC to that of bladder_ADC_/tumor_ADCmean_ and cervix_ADC_/tumor_ADCmean_ (AUC: 0.69 and 0.68, respectively) (Fig. 4b). Furthermore, myometrium_ADC_/tumor_ADCmean_ combined with FIGO stage yielded higher iAUC/AUC for predicting 5-/3-year DSS than FIGO stage alone (iAUC: 0.82 vs. 0.78, *P* = 0.02/AUC: 0.87 vs. 0.82, *P* = 0.007; Fig. 4c, d).

### Myometrium_ADC_/tumor_ADCmean_ for prognostic stratification within FIGO stages

**Fig. 5:**
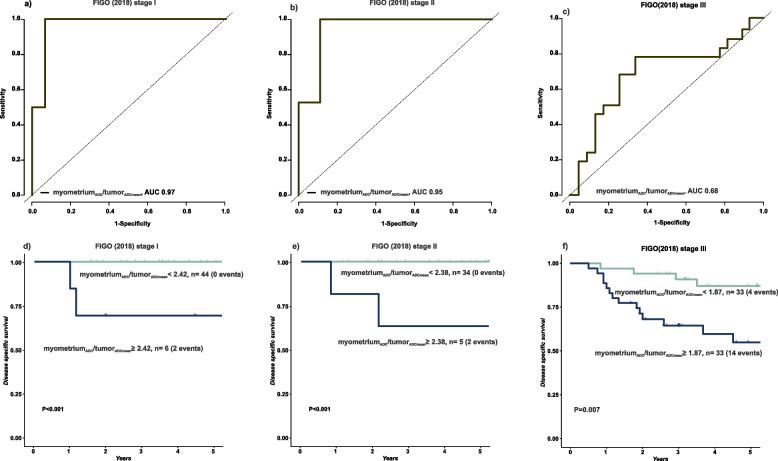
Receiver operating characteristics (ROC) curves displaying the discriminatory abilities for predicting disease-specific survival in cervical cancer within FIGO (2018) stage I (**a**), stage II (**b**), stage III (**c**) at 5 years. The ROC curves were used to calculate the Youden indexes/optimal cut-offs within FIGO (2018) stages I, II, and III for high/low-myometrium_ADC_/tumor_ADCmean_ groups. The high-myometrium_ADC_/ tumor_ADCmean_ groups had significantly lower survival than the low-myometrium_ADC_/ tumor_ADCmean_ group within FIGO (2018) stages I (**d**), stage II (**e**) and stage III (**f**). *P*-values were derived using the Log Rank test to compare survival distributions

For patient subgroups with FIGO stages I, II, and III, myometrium_ADC_/tumor_ADCmean_ yielded AUCs for the tdROC for predicting 5-year DSS of 0.97, 0.95, and 0.68, respectively (Fig. 5 a, b, c). The optimal cut-offs for myometrium_ADC_/tumor_ADCmean_ were ≥ 2.42, ≥ 2.38, and ≥ 1.87 in FIGO I, FIGO II, and FIGO III, respectively, yielding corresponding time-dependent sensitivities [specificities] of 100% [86%], 100% [90%] and 78% [63%]. FIGO I, II, and III patients with myometrium_ADC_/tumor_ADCmean_ ≥ 2.42, ≥ 2.38, and ≥ 1.87, respectively, had significantly reduced survival (Fig. 5 d, e, f). Clinical patient characteristics and MRI staging variables were mostly similar within FIGO I, FIGO II, and FIGO III when comparing patients with myometrium_ADC_/tumor_ADCmean_ ≥ / < 2.42, ≥ / < 2.38 and ≥ / < 1.87, respectively (Suppl. table 7, 8 and 9).

## Discussion

This large retrospective cohort study explored the prognostic potential of tumor ADC measurements from pre-treatment pelvic MRI in uterine cervical cancer (CC) patients. The study showed that primary tumor ADC predicted disease-specific survival (DSS) in CC. Importantly, normalizing tumor ADC by myometrium ADC yielded the best prediction of DSS compared to that of non-normalized tumor ADC. Furthermore, myometrium_ADC_/tumor_ADCmean_ yielded independent prognostic value after adjusting for the 2018 International Federation of Gynecology and Obstetrics (FIGO) stage. Importantly, this study also demonstrated good to excellent inter-reader agreement (ICC: 0.67–0.78) for the tumor ADC measurements, showcasing high reproducibility and robustness for extracting tumor ADC as a potential imaging biomarker in the clinic.

The FIGO (2018) staging system is widely used in the clinic to guide patient stratification to different treatments and follow-up regimens in CC based on risk profiles. However, some patients with low FIGO-stages (I/II) still experience recurrence and death from CC, and for FIGO stage III/IV patients, survival is also variable (5 years progression-free survival was 87%, 71%, 55%, and 16% for FIGO (2018) stages I, II, III and IV, respectively, in Grigsby et al.) [33]. Interestingly, we found that low tumor ADC and high myometrium_ADC_/tumor_ADCmean_ predicted poor outcomes in patients with FIGO-stages I (HR = 26.8, *P* = 0.01; only patients with MRI-derived maximum tumor_size_ ≥ 2 cm/FIGO stage > 1B1), II (HR = 21.5, *P* = 0.004) and III (HR = 4.01, *P* = 0.02). Importantly, the relatively few events in patients with FIGO stages I and II warrant that this finding be interpreted with care and validated in larger patient cohorts. However, myometrium_ADC_/tumor_ADCmean_ as a promising risk marker is also supported by its association with DSS for different treatment groups and FIGO stages, as well as its association with RPFS in cervical cancer.

Similarly, a recent study of 117 CC patients [34] found low whole tumor ADC (on non-normalized single slice measurements) to predict poor outcomes within FIGO stage IIIC in CC. Implementing tumor ADC in clinical risk stratification could potentially benefit high-risk patients who may be offered more aggressive treatments or frequent follow-ups while sparing low-risk patients from unnecessary interventions and associated side effects. Moreover, it may promote early detection and prevention of recurrence of CC, overall enabling more individualized treatment and better patient care. However, the clinical utility of tumor ADC hinges on its ability to provide actionable insights to clinicians and that the reported tumor ADC value can guide the selection and implementation of effective treatment strategies.

In the present study, low tumor_ADCmean_ was associated with aggressive clinical- and radiological features in CC patients. Low tumor_ADCmean_ is associated with high histologic tumor grade, confirming findings from an MRI study of 53 CC patients by Xue et al. [35]. Xue and coauthors also reported low tumor ADC to be more common in squamous cell carcinomas, which is in contrast to our study showing no significant correlation between tumor_ADCmean_ and CC histological subtype. However, this might be explained by differences in the methods used for delineation of the tumor ROIs. Whereas we intentionally drew ROIs in the areas with the most diffusion restriction for tumor_ADCmean_, Xue et al. performed 3-dimensional (3D) volumetric tumor volume delineation. Moreover, we found that tumor_ADCwhole_ was lower in squamous cell carcinomas than adenocarcinomas, supporting Xue et al.’s findings [35].

Consistency and reproducibility between readers are essential for all prognostic markers if they are to be introduced in the clinic [36]. In our study, we demonstrated high inter-reader agreement for measuring tumor ADC (ICC: 0.67–0.79), being comparable to previous reports on tumor ADC measurements in CC (ICC: 0.63–0.89) [9, 14] and endometrial cancer (ICC: 0.60) [37]. Furthermore, the inter-reader agreement for tumor ADC measurements in the present study was comparable to the agreement reported for CC MRI-assessed maximum tumor diameter (ICC of 0.73 in patients with visible tumors [38]), a metric that is incorporated in the FIGO (2018) stage assignment. Altogether, the high inter-reader agreement for tumor ADC measurements supports its potential implementation as a prognostic marker in the clinic.

Differences in MRI vendors, -protocols, -equipment, and -acquisition methodology are known to influence image quality and the extracted values for quantitative imaging markers [17, 39, 40]. It is, therefore, essential to identify MRI protocol parameters that affect the measured ADC value in the tumor and ideally correct for these. We found that several MRI protocol parameters significantly impacted the measured tumor ADC and that these effects on tumor ADC were negated by including ADC from myometrium (myometrium_ADC_) in a multivariable analysis. Moreover, we found significant correlations between the tumor ADC variables and the ADC measurement from the normal reference tissues (Spearman’s rho ≥ 0.25, *P* < 0.001), suggesting significant effects caused by differences in MRI acquisition methodology. A possible solution to limit the effect of variations in MRI protocol parameters on extracted tumor ADC values is to normalize tumor ADC with ADC measured in putative normal reference tissue [41]. Normalizing mean tumor ADC (tumor_ADCmean_) with myometrium_ADC_ by creating a ratio (myometrium_ADC_/tumor_ADCmean_) improved the prediction of DSS in the present study. Furthermore, the normalized tumor ADC marker no longer correlated to MRI protocol parameters, and the normalized ADC marker was more closely associated with other radiological, histological, and clinical risk factors. In line with this, an MRI study on 85 CC patients found that normalizing tumor ADC to urine improved prediction of recurrence-free survival [9]. Both studies support the importance of accounting for the MRI acquisition-related variation in ADC values and normalizing tumor ADC to ADC from myometrium/urine in order to improve the prognostic power of tumor ADC in CC.

Tumor ADC can be measured by delineating a large single axial tumor slice [9, 14, 42] or sampling smaller areas in one or several slices [11, 43]. We found that ROIs from several smaller tumor areas from areas with the most restricted diffusion (tumor_ADCmean_) predicted DSS stronger than ROIs from a single large tumor slice (tumor_ADCwhole_; AIC: 441 vs. 449). Our study suggests that variations in measurement methods and MRI protocols may partly explain differences in findings regarding the prognostic value of ADC in previous research. Therefore, standardizing DWI protocols and ADC measurements in CC research is essential for future consistency and reliability.

Repeated measurements of tumor ADC in multiple tumor areas with calculation of mean value might yield more robust tumor ADC estimates than measuring only one tumor area [44]. This is supported by our finding that tumor_ADCmean_ was a slightly stronger predictor of DSS than the simulated tumor ADC variable (tumor_ADCrandom_), computed by random sampling from the ten ADC measurements by the two readers (AIC: 441 vs. 444). However, we note that this difference seems marginal and that less than five measurements may be sufficient.

Consensus guidelines have recommended caution towards subjective placements of smaller ROIs [45]. However, delineating a single large axial slice in a tumor with heterogeneous diffusion restriction might not precisely capture the very high diffusion restriction areas in parts of the tumor, reducing the prognostic information from the ROI. Furthermore, even though one study [9] found lower inter-reader variability for 3-dimensional (3D) volumetric tumor volume delineation than single whole slice delineation, the 3D tumor volumetric delineation did not yield tumor ADC with superior prognostic predictions. 3D tumor segmentation is also very time-consuming and not feasible in routine clinical practice before tools for automatic 3D segmentations are available.

### Limitations

This study had some limitations. First, the pelvic MRI protocols used in this study were heterogeneous, with scanners from various vendors using different MRI acquisitions. This lack of standardization could have influenced the study results. However, one could argue that using heterogeneous data better reflects the clinical routine diagnostic setting for CC patients where different scanners are used, making the results more translatable to a routine setting. Furthermore, our findings suggest that normalizing the tumor ADC measurement to myometrium could mitigate some of the issues related to the lack of protocol standardization.

The retrospective nature of this study and its use of MRI examinations performed between 2009 and 2020 suggest that changes in imaging technologies and patient selection for MRI scans over time could have impacted the results. Furthermore, the continual evolution of MRI technology and the development of new treatment modalities for CC may supersede the immediate relevance of our current findings. Despite being one of the most extensive studies to date assessing the predictive potential of tumor ADC values in CC, this is a single-center study, and the sample size is inadequate to evaluate the prognostic impact and potential of tumor ADC within all the twelve FIGO (2018) substages. The calculated thresholds in this study are for exploratory purposes only and should not be used for clinical decision-making due to limitations in study design, power, and lack of external validation. Moreover, the study revealed a difference in average ADC values and size of the ROIs for the measurements from readers 1 and 2, potentially attributable to differences in reader experience and size of the drawn regions of interest. In spite of this, the study demonstrated overall good to excellent inter-reader agreement for the ADC measurements and comparable prognostic power of the ADC markers from the two readers.

## Conclusion

In summary, tumor ADC strongly predicted disease-specific survival in uterine cervical cancer. Normalizing tumor_ADCmean_ to myometrium_ADC_ may improve prognostication compared to using tumor_ADCmean_ alone. The ratio, myometrium_ADC_/tumor_ADCmean_, yielded the best prediction of disease-specific survival in cervical cancer and independently predicted outcomes in a multivariable Cox regression analysis, including FIGO stages. Importantly, myometrium_ADC_/tumor_ADCmean_ also yielded an independent impact on survival within the FIGO stage III, representing a promising non-invasive biomarker for improved prognostication within FIGO stage III. The value of MRI-derived pre-therapeutic tumor ADC markers should be further explored in combination with other risk markers in prospective multi-center studies and include external validation in order to assess its possible role in guiding risk-stratified primary treatment and follow-up algorithms in cervical cancer.

## Supplementary Information

Supplementary Material 1.

## Data Availability

The data used in this study is not publicly available, as it comprises sensitive patient data. Fully anonymized data may be shared by the corresponding author upon reasonable request.

## Abbreviations

ADC: Apparent diffusion coefficient
AIC: Akaike information criterion
CC: Uterine cervical cancer
DSS: Disease-specific survival
FIGO: International Federation of Gynecology and Obstetrics
iAUC: Area under the curve integrated over time
RCT: Primary radiotherapy with or without chemotherapy
RPFS: Recurrence/progression-free survival
tdROC: Time-dependent receiver operating characteristics curve
