# Supplementary material for: Tumor ADC value predicts outcome and yields refined prognostication in uterine cervical cancer
Source: Cancer Imaging. 2025 Feb 28;25:23. doi: 10.1186/s40644-025-00828-6 (PMC11871606; doi:10.1186/s40644-025-00828-6)
Supplement: Supplementary file 1 — Supplementary Material 1. [file 40644_2025_828_MOESM1_ESM.docx]

**Supplementary material**

| **Suppl. table 1. DWI-MRI protocol parameters in 179 patients with cervical cancer.** | |
| --- | --- |
| **Categorical variables** | **N(%)** |
| **Vendor** |  |
| Siemens Healthineers | 118 (66%) |
| Phillips Healthcare | 49 (27%) |
| GE Healthcare | 12 (7%) |
| **Field strength** |  |
| 1.5 Tesla | 100 (56%) |
| 3.0 Tesla | 79 (44%) |
| **Lowest b-value** |  |
| 0 | 104 (58%) |
| 50 | 75 (42%) |
| **Highest b-value** |  |
| 800 | 57 (32%) |
| 1000 | 122 (68%) |
| **Number of b-values (2–4)** |  |
| 2 | 75 (42%) |
| 3 | 69 (39%) |
| 4 | 35 (19%) |
| **Continuous variables** | **Median [IQR]** |
| Echo time [TE] (ms) | 71 [65–82] |
| Inter-slice gap (mm) | 0.60 [0.40–0.95] |
| Matrix dimension | 160 [140–256] |
| Field-of-view [FOV] (mm) | 160 [128–256] |
| Repetition time [TR] (ms) | 3600 [3100–5640] |
| Slice thickness (mm) | 4 [3–5] |
| **B-values:** measurement of the degree of diffusion weighting applied, two or more b-values is used to calculated the ADC. Lowest, highest and number of b-values used for the MRI examination is presented in the table. **DWI:** Diffusion weighted imaging. **Echo time (TE):** Time between the the radio frequency pulse and the echo signal in milli seconds. **Inter-slice gap:** Distance between MRI imaging slices. **Matrix dimension:** the number of row and columns in a MRI image slice. **Field-of-view (FOV):** Area in-plane over which the MRI image is acquired in millimeters. **Repetition time (TR):** time from the excitation pulse to the the next pulse in milliseconds. **Slice thickness:** The thickness of the image slice in millimeters. | |

| **Suppl. table 2. Inter-reader variability for apparent diffusion coefficient (ADC) value measurements by two readers in 179 patients with cervical cancer** | | | | | | | | |
| --- | --- | --- | --- | --- | --- | --- | --- | --- |
| **Variable** | **Median ADC**  **(**$\boldsymbol{\times}\boldsymbol{10}^{\boldsymbol{-6}} \mathbf{mm}^{\boldsymbol{2}}\mathbf{/}\mathbf{s}$**)** | | **Median difference**  **(**$\boldsymbol{\times}\boldsymbol{10}^{\boldsymbol{-6}} \mathbf{mm}^{\boldsymbol{2}}\mathbf{/s}$**)** | **95% limits of agreement**  $\boldsymbol{(\times}\boldsymbol{10}^{\boldsymbol{-6}} \mathbf{mm}^{\boldsymbol{2}}\mathbf{/s}$**)** | **ICC** | **Median size of ROI**  **(mm^2^) (range)** | |  |
|  | Reader 1 | Reader 2 | Reader 1/Reader2 | Reader 1/Reader2 |  | Reader 1 | Reader 2 |  |
| Tumor_ADC1_ | 704 | 648 | 90 | -121, 242 | 0.75 | 98 (5–874) | 27 (5–129) |  |
| Tumor_ADC2_ | 748 | 726 | 80 | -85, 253 | 0.71 | 90 (5–741) | 28 (5–190) |  |
| Tumor_ADC3_ | 769 | 706 | 70 | -88, 231 | 0.76 | 91 (7–951) | 25 (7–121 ) |  |
| Tumor_ADC4_ | 795 | 682 | 67 | -83, 219 | 0.77 | 80 (5–561) | 25 (5–189) |  |
| Tumor_ADC5_ | 847 | 744 | 67 | -88, 280 | 0.67 | 66 (5–597) | 25 (5–185) |  |
| Tumor_ADCmean_^a)^ | 772 | 697 | 77 | -79, 229 | 0.76 | 467(302–798) | 138 (91–189) |  |
| Tumor_ADC1whole_ | 833 | 886 | 45 | -226, 144 | 0.78 | 417 (225–770) | 631 (320–1027) |  |
| Bladder_ADC_ | 2920 | 2927 | 67 | -594, 685 | 0.79 | 536 (279–920) | 1306 (594–2282) |  |
| Cervix_ADC_ | 1642 | 1594 | 139 | -570, 585 | 0.59 | 44 (28–72) | 31 (21–47) |  |
| Myometrium_ADC_ | 1418 | 1413 | 129 | -385, 482 | 0.64 | 70 (48–109) | 61 (43–94) |  |
| Bladder_ADC_**/**tumor_ADCmean_ | 3.67 | 4.15 | 0.38 | -1.03­,1.76 | 0.74 | - | - |  |
| Cervix_ADC_**/**tumor_ADCmean_^a)^ | 2.08 | 2.20 | 0.16 | -0.60,0.97 | 0.67 | - | - |  |
| Myometrium_ADC_/tumor_ADCmean_^a)^ | 1.84 | 1.95 | 0.16 | -0.61,0.88 | 0.68 | - | - |  |
| ADC, apparent diffusion coefficient; tumor_ADC1_–tumor_ADC5_, tumor ADC values measured in five regions of interest (ROIs) in the primary tumor (presented in ascending succession: tumor_ADC1_< tumor_ADC2_ etc.), selecting the tumor areas depicting most restricted diffusion; Tumor_ADCmean_, the mean value of Tumor_ADC1_– Tumor_ADC5_; Tumor_ADCwhole_, ADC value from a ROI comprising the entire primary tumor in the slice depicting largest tumor area; Bladder_ADC_, ADC values measured in the urine in the urinary bladder; Cervix_ADC_**,** ADC values measured in the normal outer cervical stroma; Myometrium_ADC_, ADC values measured in the normal myometrium; CI, confidence interval; ICC, intra-class correlation; IQR, inter-quartile range.  ^a)^ Tumor_ADCmean_ is in this inter-reader analysis the mean of the other 5 ADC variables for each of the two readers. | | | | | | | | |

| **Suppl. table 3. Cox regression analysis including clinicopathologic- and MRI variables for predicting disease-specific survival in 179 patients with uterine cervical cancer (46 died from disease), using variables from readers 1 and 2** | | | | |
| --- | --- | --- | --- | --- |
|  | **HR** | **95%CI** | **P-value** | **AIC** |
|  | **R1/R2 ^a^** | **R1/R2 ^a^** | **R1/R2 ^a^** | **R1/R2 ^a^** |
| **Univariable Cox regression analysis** | | | | |
| Myometrium_ADC_/tumor_ADCmean_ ^a^ | 5.58/1.75 | 3.25–9.57/1.37–2.25 | <0.001/<0.001 | 422/442 |
| Age (dec) | 1.28 | 1.06–1.53 | 0.009 | 448 |
| MRI: maximum tumor size (cm) | 1.34/1.21 | 1.23–1.46/1.08–1.35 | <0.001/0.001 | 425/445 |
| MRI:invasion vagina ^b^ | 4.51/1.51 | 2.61–7.80/1.01–2.27 | <0.001/0.04 | 425/450 |
| MRI: parametrial infiltration | 2.93/0.90 | 1.05–8.16/0.42–1.94 | 0.04/0.80 | 448/443 |
| MRI: enlarged (>1 cm) lymph nodes (yes/no) | 2.03/1.69 | 1.12–3.70/0.94–3.04 | 0.02/0.08 | 449/451 |
| MRI: invasion bladder/rectum (yes/no) | 3.14/2.26 | 1.71–5.77/1.20–4.26 | <0.001/0.01 | 442/448 |
| Histologic grade (low/moderate vs. high) **^c^** | 2.90 | 1.58–5.32 | <0.001 | 444 |
| **Multivariable model stratified by FIGO(2018) stages I-IV** | | | | |
| Myometrium_ADC_/ tumor_ADCmean_ | 3.18/1.43 | 1.71–5.93/1.05–1.95 | <0.001/0.02 | - |
| MRI: maximum tumor size (cm) | 1.11/1.02 | 0.99–1.27/0.88–1.18 | 0.06/0.81 | - |
| Histologic grade (low/moderate vs. high) **^c^** | 1.83/2.43 | 1.25–4.74/ 1.22–4.86 | 0.009/0.01 | - |
| MRI:invasion vagina ^b^ | 1.87/0.87 | 0.88–3.99/0.53–1.40 | 0.10/0.53 | - |
| **Myometrium_ADC_/ tumor_ADCmean_ within FIGO(2018) stages I-IV** | | | | |
| FIGO stage I (n=50, events=3) ^d^ | 16.6/34.8 | 1.69–162/1.61–751 | 0.02/0.02 | - |
| FIGO stage II (n=39, events=4) | 53.8/19.4 | 1.61–1795/2.20–170 | 0.03/0.008 |  |
| FIGO stage III (n=66, events=20) | 7.16/3.06 | 1.84–27.8/1.02–8.88 | 0.005 /0.04 | - |
| FIGO stage IV (n=24, events=19) | 1.59/0.99 | 0.73–3.46/0.57–1.71 | 0.24/0.96 | - |
| ADC, apparent diffusion coefficient (10^-6^mm^2^/sec**)**; AIC, Akaike information criterion; FIGO, International Federation of Gynecology and Obstetrics; HR, hazard ratio.  **^a)^** Cox-regression output with variables from reader1 (R1) and reader2 (R2).  **^b)^**Ordinal variable consisting of "no invasion", "invading upper 2/3 of the vagina" and " invading lower 1/3 of the vagina".  **^c)^**Missing data were handled by multiple imputation in order to perform multivariable analysis on all patients in the sample. Data on histologic grade was missing in 11% (19/179) of the patients.  **^d)^** The study cohort included only patients with MRI derived maximum tumor size ≥ 2 cm, consequently there were only FIGO stage ≥ 1B2 in the analysis in FIGO stage I. | | | | |

| **Suppl. table 4: Myometrium_ADC/_tumor_ADCmean_ values in relation to FIGO (2018) stage, pelvic tumor extent (from MRI) and histologic subtype/grade** | | | | | |
| --- | --- | --- | --- | --- | --- |
| **Variable** | **N: 179**  **(100%)^(a)^** | **MyometriumADC/tumorADCmean** | | **P-value** | |
| **FIGO (2018) stage** |  |  | | <0.001 | |
| I | 50 (28%) | 1.74 [1.58–2.00] | |  | |
| II | 39 (22%) | 1.79 [1.63–2.01] | |  | |
| III | 66 (37%) | 1.92 [1.68–2.17] | |  | |
| IV | 24 (13%) | 2.12 [1.93–2.68] | |  | |
| **MRI: Vaginal invasion** |  |  | | <0.001 | |
| No | 49 (28%) | 1.73 [1.53–1.93] | |  | |
| Upper two thirds | 119 (67%) | 1.94 [1.71–2.18] | |  | |
| Lower third | 11 (6%) | 1.98 [1.81–2.58] | |  | |
| **MRI: Parametrial invasion** |  |  | | 0.02 | |
| No | 43 (24%) | 1.74 [1.53–1.96] | |  | |
| Yes | 136 (76%) | 1.93 [1.70–2.17] | |  | |
| **MRI: Enlarged (>1 cm)** **lymph nodes** |  |  | | 0.02 | |
| No | 137 (77%) | 1.84 [1.64–2.09] | |  | |
| Yes | 42 (23%) | 2.05 [1.71–2.32] | |  | |
| **MRI: Invasion into rectum/urinary bladder** |  |  | | 0.05 | |
| No | 133 (74%) | 1.82 [1.62–2.15] | |  | |
| Yes | 36 (26%) | 1.99 [1.78–2.22] | |  | |
| **Histologic subtype** |  |  | | 0.14 | |
| Adenocarcinoma | 33 (18%) | 1.73 [1.49–2.17] | |  | |
| Squamous cell carcinoma | 139 (78%) | 1.91 [1.71–2.12] | |  | |
| Other | 7 (4%) | 2.66 [1.64–3.17] | |  | |
| **Histologic grade** |  |  | | <0.001 | |
| Low/moderate grade | 129 (80%) | 1.85 [1.67–2.10] | |  | |
| High grade | 32 (20%) | 2.10 [1.82–2.65] | |  | |
| **MyometriumADC/tumorADCmean as dependent variable** | | | | | |
| **Variable** | | **R^2^** | **β** | | **P** |
| **Linear regression for continuous variables ^(b)^** | | | | | |
| **Age (pr. decade) (n=179), median (IQR): 49 (39–62)** | | 0.00 | 0.01 | | 0.52 |
| **MRI: Maximum tumor size diameter (cm) (n=179), median (IQR): 4.8 (3.5–6.2)** | | 0.06 | 0.04 | | <0.001 |
| P-values represent the difference in myometriumADC/tumorADCmean across groups, estimated by Mann Whitney U test and by Kruskal Wallis test, Joncheere Terpsta trend test for multiple categories, or a significant association between myometriumADC/tumorADCmean and an independent variable in linear regression.  ADC = apparent diffusion coefficient; CCRT = Concurrent radiation and chemotherapy; Grade 1 = low grade/well differentiated; Grade 2 = intermediate grade/moderately differentiated; Grade 3 = High grade/poorly differentiated; IQR, inter-quartile range; FIGO = International Federation of Gynecology and Obstetrics; RT = Radiotherapy  ^(a)^ Complete data for 179 patients except histologic grade (n=161).  ^(b)^Linear regression analysis with myometriumADC/tumorADCmean as the dependent variable. | | | | | |

| **Suppl. table 5. Pelvic DWI-MRI protocol parameters and ADC of reference tissue in relation to tumor_ADCmean_ (10^-6^ S/mm^2^) in cervical cancer (n=179)** | | | | | | |
| --- | --- | --- | --- | --- | --- | --- |
|  | **Univariable beta** | **R^2^** | **P ^a)^** | **Multivariable ^b)^ beta** | **Model R^2 c)^**  **: 0.17** | **P ^a)^** |
| **DWI-MRI protocol variables** |  | | | | | |
| Vendor **^d)^** | 3.4 | 0.00 | 0.84 | - | - | - |
| Field-strength (1.5 T vs. 3.0 T) | 63.7 | 0.05 | 0.002 | 41.4 | - | 0.17 |
| Field-of-view [FOV] (mm) | -19.6 | 0.00 | 0.44 | - | - | - |
| Matrix dimension | 0.0 | 0.00 | 0.97 | - | - | - |
| Slice thickness (mm) | 7.0 | 0.00 | 0.51 | - | - | - |
| Inter-slice gap (mm) | -17.4 | 0.00 | 0.57 | - | - | - |
| Echo time [TE] (ms) | -1.0 | 0.00 | 0.34 | - | - | - |
| Repetition time [TR] (ms) | 0.02 | 0.03 | 0.02 | 0.0 | - | 0.38 |
| Lowest b-value(0 vs. 50) | -1.1 | 0.04 | 0.009 | 0.02 | - | 0.98 |
| Highest b-value(1000 vs. 800) | 0.2 | 0.01 | 0.12 | - | - | - |
| Number of b-values (2–4) | -23.7 | 0.02 | 0.07 | - | - | - |
| **ADC values from normal reference tissue** |  | | | | | |
| Bladder_ADC_ (pr. 10 unit increase) | 0.9 | 0.10 | <0.001 | 0.1 | - | 0.75 |
| Cervix_ADC_ (pr. 10 unit increase) | 1.6 | 0.10 | <0.001 | 0.1 | **-** | 0.80 |
| Myometrium_ADC_ (pr. 10 unit increase) | 2.2 | 0.14 | <0.001 | 1.9 | **-** | 0.01 |
| ADC, apparent diffusion coefficient; Bladder_ADC_, ADC values measured in the urine in the urinary bladder; Cervix_ADC_**,** ADC values measured in the normal outer cervical stroma; DWI, diffusion weighted imaging; Echo time (TE), time from the center of the MRI-radio frequency-pulse to the center of the echo-signal; ms, milli-seconds; Field-of-view [FOV], Area in-plane over which the MRI image is acquired in millimeters; Myometrium_ADC_, ADC values measured in the normal myometrium; R^2^, the proportion of the variance of the dependent variable that is predictable from the explaining variable; Repetition time(TR), the time interval between the time point of repeating series of MRI-pulse and echo; Tesla(T), Magnetic Field Intensity Unit.  **^a)^** Linear regression analysis of variable relations to tumor_ADCmean_.  **^b)^** Significant variables in the univariable model were included in the multivariable model.  **^c)^** R^2^-value represents the explained variance of tumor_ADCmean_ by all variables together in the multivariable model.  **^d)^** Vendor was treated as a categorical variable comparing the MRI-vendors Siemens Healthineers, Phillips Healthcare, and GE Healthcare | | | | | | |

| **Suppl. table 6. Multivariable Cox regression analysis including clinicopathologic- and MRI variables for predicting recurrence or progression in 160 patients with uterine cervical cancer in FIGO stage IB2–IVA (40 had recurrence or progression of the disease)** | | | |
| --- | --- | --- | --- |
|  | **HR** | **95%CI** | **P^a^** |
| **Multivariable model** | | | |
| Myometrium_ADC_/tumor_ADCmean_ | 2.29 | 1.12–4.69 | 0.02 |
| MRI: maximum tumor_size_ (cm) | 1.26 | 1.06–1.50 | 0.01 |
| MRI: invasion vagina^b^ | 1.77 | 0.77–4.08 | 0.17 |
| Histologic grade (low/moderate vs.high)^c^ | 3.11 | 1.57–6.16 | 0.001 |
| **Multivariable model stratified by FIGO (2018) stages I-IV** | | | |
| Myometrium_ADC_/tumor_ADCmean_ | 4.80 | 1.23–6.01 | 0.01 |
| MRI: maximum tumor_size_ (cm) | 1.28 | 1.06–1.56 | 0.02 |
| MRI: invasion vagina^c^ | 1.85 | 0.70–4.92 | 0.22 |
| Histologic grade (low/moderate vs.high)^d^ | 2.60 | 1.28–5.31 | 0.009 |
| **Myometrium_ADC_/tumor_ADCmean_ in FIGO (2018) stages I-IV** | | | |
| FIGO stage I (n=50, events=8)^d^ | 11.1 | 2.83–43.1 | <0.001^e^ |
| FIGO stage II (n=39, events=4) | 47.8 | 4.68–177 | 0.001 ^e^ |
| FIGO stage III (n=66, events=24) | 5.34 | 2.09–13.7 | <0.001^e^ |
| **Myometrium_ADC_/tumor_ADCmean_ in treatment groups** | | | |
| Surgery (n=27, events=2) | 10.0 | 1.15–87.7 | 0.04 |
| Surgery & adjuvant therapy (n=16, events=6) | 4.07 | 0.89–18.7 | 0.07 |
| RCT (n=117, events=32) | 3.75 | 1.51–9.29 | <0.001 |
| ADC, apparent diffusion coefficient (10^-6^mm^2^/sec**)**; AIC, Akaike information criterion; CI, confidence interval; FIGO, International Federation of Gynecology and Obstetrics; HR, hazard ratio; RCT: Primary radiotherapy with or without chemotherapy.  **^a^** Cox regression analysis  **^b^** Ordinal variable consist of "no invasion", "invading upper 2/3 of the vagina" and "invading lower 1/3 of the vagina".  **^c^** Missing data were handled by multiple imputation in order to perform multivariable analysis on all patients in the sample. Data on histologic grade was missing in 11% (19/179) of the patients.  ^d^ The study cohort included only patients with MRI derived maximum tumor size ≥ 2 cm, consequently there were only FIGO stage ≥ 1B2 in the analysis in FIGO stage I.  ^e^ Myometrium_ADC_/tumor_ADCmean_ remained significant also after adjusting for MRI: maximum tumor_size_ in FIGO (2018) I, II and III (P=0.001, P=0.006 and P=0.004, respectively). | | | |

| **Suppl. table 7. Comparison of clinical and MRI staging variables in patients with high and low myometrium_ADC_/tumor_ADCmean_ within FIGO(2018) stage I ^a)^ in cervical cancer** | | | |
| --- | --- | --- | --- |
| **Variable** | **Low value^b^**  **(n_1_=44)** | **High value^b^**  **(n_2_=6)** | **P** |
| **Age (yrs.)** |  |  | 0.25 ^c^ |
| Median (IQR) | 41 (36–55) | 53 (41–59) |  |
| **MRI: maximum tumor size (cm)** |  |  | 0.28 ^c^ |
| Median (IQR) | 3.3 (2.6–3.9) | 2.7 (2.3–3.2) |  |
| **Primary**  **treatment** |  |  | 0.84 ^d^ |
| Surgery only | 22 | 4 |  |
| Surgery and adjuvant treatment | 7 | 1 |  |
| Primary radiotherapy with or without chemotherapy | 15 | 1 |  |
| Other | 0 | 0 |  |
| **Histologic subtype** |  |  | 0.54 ^d^ |
| Adenocarcinoma | 12 | 1 |  |
| Squamous cell carcinoma | 30 | 4 |  |
| Other | 2 | 1 |  |
| **Histologic grade (low/moderate vs.high) (n_1_=42, n_2_=5)** |  |  | 0.51 ^d^ |
| Low/moderate | 37 | 4 |  |
| High | 5 | 1 |  |
| ADC, Apparent diffusion coefficient; FIGO, International Federation of Gynecology and Obstetrics; IQR, inter-quartile range  ^a^ The study cohort included only patients with MRI derived maximum tumor size ≥ 2 cm, consequently there were only FIGO stage ≥ 1B2 in the analysis in FIGO stage I..  ^b^ The optimal cut-offs for myometrium_ADC_/tumor_ADCmean_ for predicting 5-year disease-specific survival in FIGO I were 2.42 with values ≥ defining high risk and < defining low risk.  ^c^ Mann–Whitney U test  ^d^ Pearson`s chi-square test | | | |

| **Suppl. table 8. Comparison of clinical and MRI staging variables in patients with high and low myometrium_ADC_/tumor_ADCmean_ within FIGO(2018) stage II in cervical cancer** | | | |
| --- | --- | --- | --- |
| **Variable** | **Low value^a)^**  **(n_1_=34)** | **High value^a)^**  **(n_2_=5)** | **P** |
| **Age (yrs.)** |  |  | 0.67 ^b)^ |
| Median (IQR) | 41 (36–55) | 44 (40–54) |  |
| **MRI: maximum tumor size (cm)** |  |  | 0.25 ^b)^ |
| Median (IQR) | 4.9 (4.4–5.6) | 4.4 (4.1–4.6) |  |
| **MRI: parametrial infiltration** |  |  | 0.44 ^c)^ |
| No | 3 | 1 |  |
| Yes | 31 | 4 |  |
| **MRI: invasion vagina** |  |  | 1.0 ^c)^ |
| no | 8 | 1 |  |
| Upper two thirds | 26 | 4 |  |
| Lower third | 0 | 0 |  |
| **Primary**  **treatment** |  |  | 0.78 ^c)^ |
| Surgery only | 1 | 0 |  |
| Surgery and adjuvant treatment | 2 | 0 |  |
| Primary radiotherapy with or without chemotherapy | 31 | 5 |  |
| Other | 0 | 0 |  |
| **Histologic subtype** |  |  | 0.17 ^c)^ |
| Adenocarcinoma | 7 | 3 |  |
| Squamous cell carcinoma | 26 | 2 |  |
| Other | 1 | 0 |  |
| **Histologic grade (low/moderate vs.high) (n_1_=28, n_2_=5)** |  |  | 0.08 ^c)^ |
| Low/moderate | 25 | 2 |  |
| High | 4 | 3 |  |
| ADC, Apparent diffusion coefficient; FIGO, International Federation of Gynecology and Obstetrics; IQR, inter-quartile range  a) The optimal cut-offs for myometrium_ADC_/tumor_ADCmean_ for predicting 5-year disease-specific survival in FIGO I were 2.38 with values ≥ defining high risk and < defining low risk.  ^b)^ Mann–Whitney U test  ^c)^ Pearson`s chi-square test | | | |

| **Suppl. table 9. Comparison of clinical and MRI staging variables in patients with high ^a)^ and low ^a)^ myometrium_ADC_/tumor_ADCmean_ within FIGO (2018) stage III in cervical cancer** | | | |
| --- | --- | --- | --- |
| **Variable** | **Low value**  **(n_1_=33)** | **High value**  **(n_2_=33)** | **P** |
| **Age (yrs.)** |  |  | 0.92 ^b)^ |
| Median (IQR) | 50 (37–59) | 49 (39–59) |  |
| **MRI: maximum tumor size (cm)** |  |  | 0.07 ^b)^ |
| Median (IQR) | 5.2 (4.7–6.0) | 6.3 (4.8–6.8) |  |
| **MRI: parametrial infiltration** |  |  | 1.0 ^c)^ |
| No | 2 | 2 |  |
| Yes | 31 | 31 |  |
| **MRI: invasion vagina** |  |  | 0.31 ^c)^ |
| no | 5 | 2 |  |
| Upper two thirds | 27 | 28 |  |
| Lower third | 1 | 3 |  |
| **MRI: Enlarged lymphnodes (>1 cm),** |  |  | 1.00 ^c)^ |
| No | 21 | 20 |  |
| Yes | 12 | 13 |  |
| **Primary**  **treatment** |  |  | 0.23 ^c^ |
| Surgery only | 0 | 0 |  |
| Surgery and adjuvant treatment | 5 | 1 |  |
| Primary radiotherapy with or without chemotherapy | 28 | 31 |  |
| Other | 0 | 1 |  |
| **Histologic subtype** |  |  | 0.60 ^c)^ |
| Adenocarcinoma | 5 | 5 |  |
| Squamous cell carcinoma | 27 | 28 |  |
| Other | 1 | 0 |  |
| **Histologic grade (low/moderate vs.high) (n_1_=26, n_2_=30)** |  |  | 0.18 ^c)^ |
| Low/moderate | 23 | 21 |  |
| High | 3 | 9 |  |
| ADC, Apparent diffusion coefficient; FIGO, International Federation of Gynecology and Obstetrics; IQR, inter-quartile range  a) The optimal cut-offs for myometrium_ADC_/tumor_ADCmean_ for predicting 5-year disease-specific survival in FIGO III were 1.87 with values ≥ defining high risk and < defining low risk.  ^b)^ Mann–Whitney U test  ^c)^ Pearson`s chi-square test | | | |
